# Significant Correlation Between Overall Survival and Mean Lung Dose in Lung Stereotactic Body Radiation Therapy (SBRT)

**DOI:** 10.3389/fonc.2020.01577

**Published:** 2020-08-11

**Authors:** Guillaume Dupic, Julian Biau, Ioana Molnar, Vincent Chassin, Véronique Dedieu, Michel Lapeyre, Aurélie Bellière-Calandry

**Affiliations:** ^1^Department of Radiation Oncology, University of Clermont Auvergne, Jean Perrin Center, Clermont-Ferrand, France; ^2^INSERM U1240 IMoST, University of Clermont Auvergne, Clermont-Ferrand, France; ^3^Department of Clinical Research UMR 501, Jean Perrin Center, Clermont-Ferrand, France; ^4^Department of Medical Physics, University of Clermont Auvergne, Jean Perrin Center, Clermont-Ferrand, France

**Keywords:** lung, stereotactic radiotherapy, prognostic factor, mean lung dose, toxicity

## Abstract

**Background:**

After stereotactic body radiation therapy (SBRT) for medically inoperable stage I non-small-cell lung cancer (NSCLC), more patients die of comorbidities, particularly severe pulmonary insufficiency, than of tumor progression. The aim of this study was to evaluate correlation between lung biologically effective dose (BED) with an α/β ratio of 3 Gy (BED_3_) and overall survival (OS) for these patients.

**Methods:**

From 2012 to 2017, we have developed a prospectively updated institutional database for all first 100 consecutively treated patients with inoperable Stage 1 (T1T2N0M0) NSCLC. All SBRT were conducted on a Novalis Tx^®^ LINAC with two coplanar dynamic conformal arcs (84%) or with coplanar volumetric modulated arc therapy (VMAT) (16%). Mean GTV and PTV were 8.6 cc and 50.8 cc, respectively. The marginal dose prescribed to the PTV was the 80% isodose line (IDL), i.e., 54 Gy in 3 fractions for 76 patients (BED_10_ = 126 Gy) and 50 Gy in 5 fractions for 24 patients (BED_10_ = 83.3 Gy). Pulmonary heterogeneity has been taken into account by using Monte Carlo or AAA algorithms. Median follow-up was 25 months.

**Results:**

At 1, 2, 3 and 5 years, local control (LC) was respectively 100, 98.2, 98.2, and 77.7%, and OS was respectively 83, 71.2, 58.1, and 33.2% (median OS was 49 months). Significant OS prognostic factors in univariate and multivariate analysis were mean lung BED_3_ (HR = 1.14, *p* = 0.01) and PTV volume (HR = 1.01, *p* = 0.004). A mean lung BED_3_ ≤ 5 Gy was significantly associated with a doubling of median OS from 29 months to more than 60 months (not achieved, *p* = 0.0068). For patients with a forced expiratory volume in 1 second (FEV1) ≤ 40%, a mean lung BED_3_ ≤ 4 Gy was significantly associated with a doubling of median OS from 23 to 46 months (*p* = 0.019).

**Conclusion:**

Mean lung BED_3_ is strongly and significantly associated with OS in SBRT for inoperable Stage I NSCLC. For all treated patients, a mean lung BED_3_ ≤ 5 Gy lead to a doubling of median OS. This threshold value should be reduced to 4 Gy for patients with FEV1 ≤ 40%.

## Highlights

- Grade 5 radiation pneumonitis after lung SBRT is probably under-evaluated because of poor baseline pulmonary function of treated patients.
- Mean lung BED_3_ is a strong and significant prognostic factor of overall survival after lung SBRT.
- Mean lung BED_3_ ≤ 5 Gy (i.e., 3.6 Gy in 3 fractions or 4.3 Gy in 5 fractions) is significantly associated with higher overall survival after lung SBRT with a doubling in median overall survival.
- For patients with poor baseline pulmonary function (FEV1 or DLCO ≤ 40%), this threshold should be reduced to 4 Gy (i.e., 3 Gy in 3 fractions or 3.5 Gy in 5 fractions).

## Introduction

Stereotactic body radiation therapy (SBRT) is the standard treatment for medically inoperable stage I non-small-cell lung cancer (NSCLC) (1). Generally, overall survival (OS) for these patients in prospective studies is around 81–100, 65–70, 39–68, and 30–65%, respectively at 1, 2, 3 and 5 years. Local control (LC) is high, around 86–100 and 79–85%, respectively at 3 and 5 years (1–3). It is widely agreed that more NSCLC patients treated with SBRT die of comorbidities than of tumor progression. Significant prognostic factors are age (4), gender (5), performance status (4, 6), histologic type (4), tumor volume (5–7), pretreatment maximum standardized uptake value (SUV_max_) (6, 8, 9), platelet-to-lymphocyte ratio (8, 10), pretreatment immune parameters (neutrophil-to-lymphocyte ratio, neutrophil and lymphocyte counts) (10) and prescribed dose (11). The role of lung dosimetric parameters as prognostic factors remains unknown. After lung SBRT, many patients died from severe pulmonary insufficiency attributed to their previous medical history [OS at 5 years is 30–65% after SBRT vs. 60–80% after surgery (12)], which makes severe radiation-induced pneumonitis (RP) difficult to interpret (2, 13).

RP is the most frequent complication after lung SBRT (14). Clinically symptomatic RP seems to develop mostly in 10–20% of patients (range: 0–49% among published studies) with most patients having asymptomatic Grade 1 pneumonitis (2, 15–17). Pre-treatment pulmonary function tests have not been shown to be predictive for RP. Therefore, patients with NSCLC with a poor baseline pulmonary function are not excluded from treatment with SBRT (18). Mean lung dose (MLD) seems to be a strong and reproducible dosimetric parameter of RP, with a significant cut-off at 4–4.7 Gy in three fractions, and is often correlated to the volume of PTV (15, 17, 19–21). Many factors may have confounded the reported MLD because of inadequate heterogeneity correction algorithms, various dose prescriptions and fractionations and probably a lack of lung volume definitions (whole or ipsilateral lung volume minus GTV or PTV) (17, 22, 23). In this way, the biologically effective dose (BED) determined with adequate heterogeneity correction algorithms may be used for estimating toxicity probabilities. For high fraction doses, the linear-quadratic model with an α/β ratio of 3 Gy is the best method for converting the physical lung dose to predict RP (24).

Our study aimed to evaluate the correlation between lung BED_3_ and OS in a prospectively updated institutional cohort of 100 consecutively treated patients with stage I NSCLC. The secondary objective was to study the impact of lung dosimetric parameters in a population with a poor baseline pulmonary function.

## Patients and Methods

### Patients’ Selection and Characteristics

From October 2012 to August 2017, we have developed a prospectively updated institutional database for all patients consecutively treated with SBRT for inoperable Stage 1 (T1T2N0M0) NSCLC in our institution. This database has been approved by our local ethics committee and a regional ethics committee (CECIC Rhône-Alpes-Auvergne, Grenoble, IRB 5921), and developed according to the French law regulating clinical research (Loi Huriet). Thus, our study is an observational cohort study from all first 100 patients recorded in this prospectively updated institutional database. All treated patients fulfilled inclusion criteria of those first described by Timmerman et al. in the analysis of RTOG 0236 (25): performance status (PS) ≤ 2, age ≥ 18 years, stages T1T2N0M0, peripherally located NSCLC at least 2 cm from the proximal bronchial tree, and medical inoperability (baseline forced expiratory volume in one second (FEV1) ≤ 40%, predictive postoperative FEV1 ≤ 30%, diffusing capacity for carbon monoxide (DLCO) ≤ 40%, severe cerebral, pulmonary or cardiovascular disease or patient refusal).

All patients were required to have a complete imaging screening performed less than 1 month prior to lung SBRT: a high-resolution contrast-enhanced lung computed tomography (CT), a 18F-fluorodeoxyglucose positron emission tomography (18F-FDG PET) and a cerebral magnetic resonance imaging (MRI) to exclude regional or distant metastases. A flexible bronchoscopy was needed to exclude an endobronchial location or infectious disease such as lung tuberculosis. A cytologically or histologically proven NSCLC was strongly recommended but not mandatory in case of contraindications. If this proof was not obtained, a tumor growth observed with an interval at least 3 months between two CT or a maximum standardized uptake value (SUV_max_) in 18F-FDG PET above 2.5–3 was necessary to include patients. Exclusion criteria were small-cell lung cancer, mediastinal location and no meeting of normal tissue dose constraints. Pre-treatment pulmonary function tests were performed for all patients.

All pre-treatment characteristics of the 100 included patients are reported in Table 1. Most patients were elderly [median age = 70 years (range: 47–90)] and male (79%) with a good general status (93% PS ≤ 1) and no history of lung surgery (78%) or radiotherapy (93%), but with poor baseline pulmonary function [57% NYHA (New York Heart Association) class ≥ 2 dyspnea, median FEV1 was 62% (20–100%) and median DLCO was 48% (8–100%)]. Fifty-six percent of patients had no histologically or cytologically proven NSCLC. Mean GTV was 8.6 cc (0.2–61.5 cc), i.e., about 2 cm in diameter.

**TABLE 1:** Patients and SBRT characteristics.

Characteristics	Number
**Patients’ characteristics**	
Total	100(100%)
Gender	
Female	21(21%)
Male	79(79%)
Age (years)	
Mean	71(47.1−90.4)
Performance status	
0	54(54%)
1	39(39%)
2	7(7%)
≥3	0(0%)
Medical history	
Surgery	
Pneumonectomy	4(4%)
Lobectomy	18(18%)
None	78(78%)
Lung radiotherapy	
Yes	7(7%)
No	93(93%)
Pre-treatment dyspnea (NYHA)	
0	18(18%)
1	25(25%)
2	18(18%)
3	30(30%)
4	9(9%)
Pre-treatment pulmonary function	
FEV1(%)	
Mean	64.1(20−100)
DLCO (%)	
Mean	49.2(8.0−100)
**Characteristics of pulmonary nodules**	
Histological/cytological proof	
None	56(56%)
Adenocarcinoma	32(32%)
Squamous cell carcinoma	9(9%)
Undifferentiated carcinoma	1(1%)
Neuroendocrine carcinoma	2(2%)
RTOG localization	
Central	7(7%)
Peripheral	93(93%)
Pulmonary localization	
Right upper lobe	36(36%)
Right middle lobe	5(5%)
Right lower lobe	14(14%)
Left upper lobe	31(31%)
Left lower lobe	14(14%)
Pre-treatment SUVmax	
Mean	7.5(1.2−19.5)
Tumor volume	
Longest diameter (mm)	
Mean	23.4(7.8−53.9)
GTV (cc)	
Mean	8.6(0.2−61.5)
PTV (cc)	
Mean	50.8(3.8−223.1)
**SBRT characteristics**	
Technique	
DCA	84(84%)
VMAT	16(16%)
Fractionation	
3	76(76%)
5	24(24%)
Overall treatment time (days)	
Mean	8(4−35)
Isocenter prescribed BED_10_ (Gy)	
Mean	196(100−219)
Received PTV BED_10_ (Gy)	
D_max_	198(87−252)
D_2 %_	194(87−246)
D_98 %_	137(111−165)
D_min_	110(27−166)
80% prescription isodose volume (%)	79(39.8−100)
Treatment quality	
Conformality index (CI)	1.19(1.0−1.64)
R50	1.8(0.46−5.72)
D2cm (Gy)	36.6(22.7−57.5)
**Follow-up (months)**	
Mean	27(0.6−64)
Median	25(0.6−64)

*NHYA: New York Heart Association, SUVmax: maximum Standardized Uptake Value, FEV1: Forced Expiratory Volume in 1 second, DLCO: Diffusing Lung capacity for Carbon Monoxide, DCA: Dynamic Conformal Arcs, VMAT: volumetric modulated arc therapy, BED10 = biologically effective dose with an α/β ratio of 10 Gy, Conformality Index (CI): ratio of 80% prescription isodose volume to the PTV, R50: ratio of 50% prescription isodose volume to the PTV, D2cm: maximum dose 2 cm from the PTV in any direction.*

### SBRT Specifications

The gross tumor volume (GTV) was contoured on 2.5-mm-thick lung CT windows. Intrafraction tumor motion due to breathing was limited by an abdominal compression and taken into account by creating an internal target volume (ITV) obtained with a four-dimensional (4D) CT scan at the time of CT simulation. An additional margin of 8 mm for adenocarcinoma and 6 mm for other histologic types was added for microscopic tumor extension to create the clinical target volume (CTV) (26, 27). Finally, the planning target volume (PTV) was obtained with a uniform 3 mm CTV expansion, according to our defined geometric stereotactic conditions.

All SBRT treatment characteristics are reported in Table 1. The isocenter prescription dose was 67.5 Gy in 3 fractions and reduced to 62.5 Gy in 5 fractions for central lung tumors to meet normal tissue dose contraints. The marginal isodose line prescribed to the edge of PTV was the 80% isodose: respectively, 54 Gy in 3 fractions and 50 Gy in 5 fractions (BED_10_ = 126 Gy and 83.3 Gy). Each fraction was separated by at least 40 h (25).

All treatments used 6-MV photons. Dose distributions were performed with two coplanar dynamic conformal arcs (DCA) in 84% of cases and with volumetric modulated arc therapy (VMAT) in 16%. Treatment planning systems (TPS) were Iplan^®^ v4.1 (Brainlab, Feldkirchen, Germany) for DCA plans and Eclipse^®^ v13.5 (Varian Medical Systems, Palo Alto, CA, United States) for VMAT plans. Pulmonary heterogeneity has been taken into account by using the Monte Carlo algorithm for DCA plans and the AAA algorithm for VMAT plans. Target coverage was adequate when at least 95% of the PTV was covered by 80% of the prescribed isodose. Treatment quality was verified by calculating the conformality index (CI) (a ratio of 80% prescription isodose volume to the PTV), a ratio of 50% prescription isodose volume to the PTV (R50) and the maximum dose 2 cm from the PTV in any direction (D2cm), as seen in Table 1 (28). Lung SBRT was performed using a Novalis Tx^®^ (Varian Medical Systems, Palo Alto, CA, United States) linear accelator (LINAC) with an integrated Exactrac X-ray 6D system^®^ (Brainlab AG, Feldkirchen, Germany). This system enabled a pre-treatment positioning which was then adjusted daily with a Cone Beam CT (CBCT).

### Follow-Up

Follow-up included prospective clinical examination and CT scans every 3 months during the first two years post-SBRT and every 6 months afterward. Follow-up PET scans were required only in cases of progressive soft tissue abnormalities observed on CT. Efficacy was assessed with the Response Evaluation Criteria in Solid Tumors (RECIST) (29). A complete response was defined as the disappearance of the target lesion, a partial response as a decrease of at least 30% of the tumor’s longest diameter and a progressive disease as an increase of at least 20% of the longest diameter. LC was defined as the absence of local failure. Local failure was characterized as the combination of a RECIST progressive disease and evidence of tumor viability as shown by biopsy or SUV_max_ in 18F-FDG PET above the pre-treatment SUV_max_ or above a value of 5 (30). Progression-free survival (PFS) was defined as the period of time from the end of SBRT to the date of local-regional failure, disseminated (visceral or lymph-node) recurrence or the patient’s death. OS was defined as the time between the end of SBRT and the patient’s death. Toxicity was evaluated with the National Cancer Institute’s Common Toxicity Criteria for Adverse Events version 4.0 (NCI-CTCAE).

Mean and median follow-up were respectively 27 and 25 months (range: 0.6–64). Only one patient was lost to follow-up after 10 months (Table 1).

### Statistical Analysis

**FIGURE 1:**
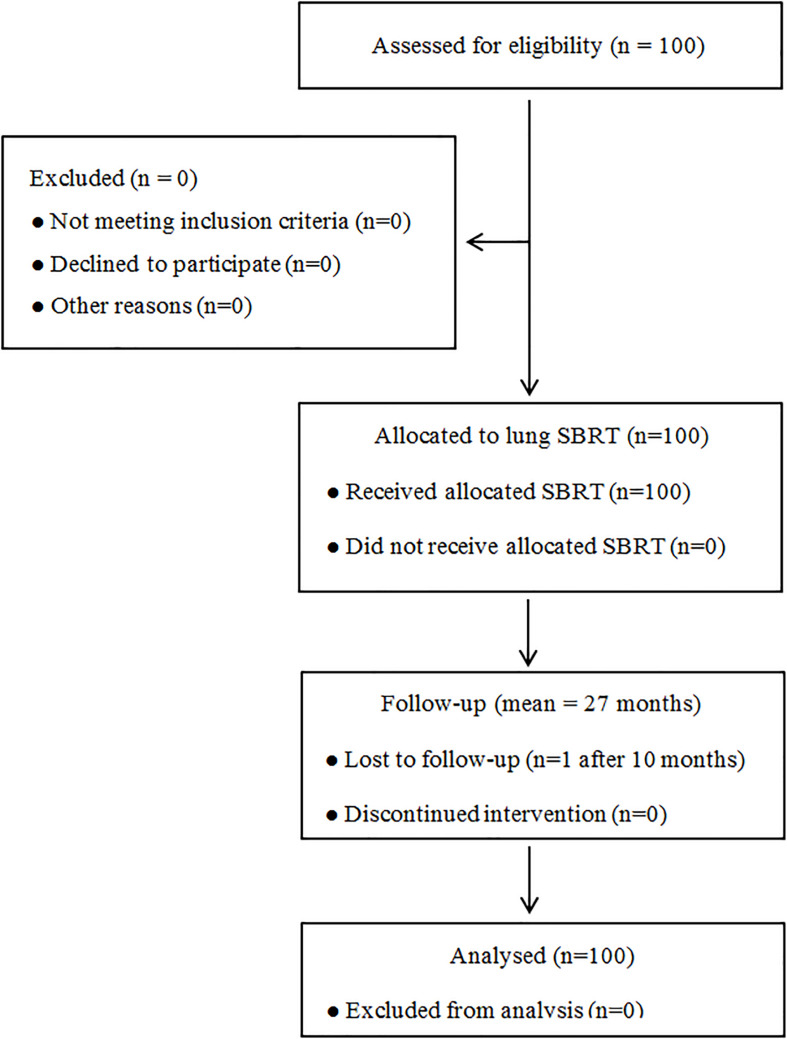
Flowchart of the patient enrollment process of study cohort.

No patient included in the prospectively updated institutional database of all first 100 patients consecutively treated with SBRT in our institution was excluded from the study or from the statistical analysis (Figure 1). LC, PFS, and OS were calculated using the Kaplan–Meier method. The Cox proportional hazards model was performed to identify predictive factors of LC and prognostic factors of PFS and OS. A two-sided *p*-value < 0.05 was considered significant. The following factors were included in the univariate analysis for LC: histological type, pre-treatment SUV_max_, conformality index, R50, GTV, PTV, an 80% prescription isodose volume (%), and maximum and minimum PTV BED_10_ (D_max_, D_98 %_, D_2 %_, D_min_). Concerning PFS and OS, the following factors were included in the univariate analysis in addition to the previously studied LC predictive factors: gender, age, performance status, history of lung surgery, pre-treatment FEV1, pre-treatment DLCO, baseline pulmonary function, and mean lung BED_3_ (whole lungs, ipsilateral lung, whole lungs minus PTV, ipsilateral lung minus PTV), The Benjamini–Hochberg method was used to adjust *p*-values to limit false positives, considering the large number of tests. The Spearman correlation enabled the identification of strongly correlated factors between them that were not included in the multivariate analysis (Spearman’s rank correlation coefficient of 0.75). Factors associated with a *p*-value < 0.25 in the univariate analysis were included in the multivariate analysis if they were also selected by the LASSO method. The Wald test and the Likelihood ratio test were performed to calculate and verify the *p*-value for each coefficient in multivariate analyses.

Linear correlation between OS and significant prognostic factors was then verified with a Pearson or Spearman correlation coefficient, depending on the cases, especially for patients with a poor pre-treatment pulmonary function (i.e., FEV1 or DLCO ≤ 40%). For each significant linear correlation observed, a ROC (Receiver Operating Characteristics) curve was performed to identify the best threshold. Finally, comparisons of OS curves with obtained thresholds were conducted using the log-rank test.

## Results

### Local Control and Progression-Free Survival

**FIGURE 2:**
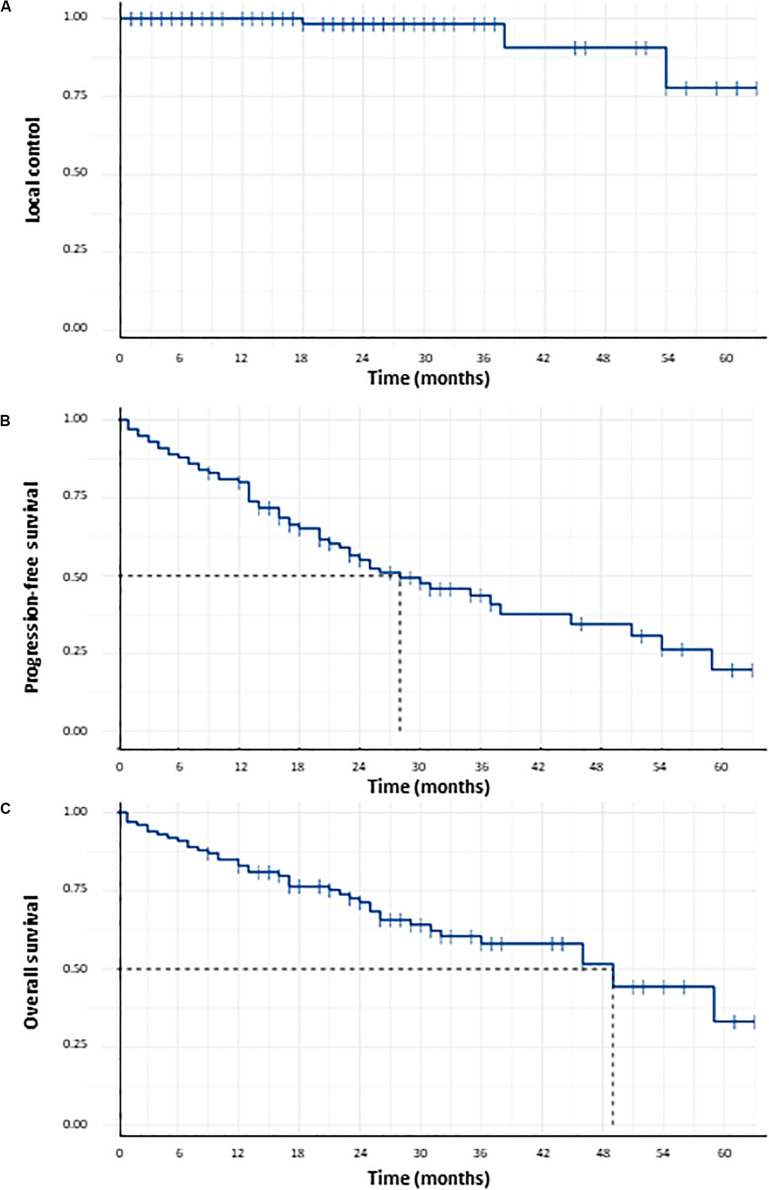
Probability of local control **(A)**, probability of progression-free survival **(B)** and probability of overall survival **(C)** for the 100 patients receiving lung SBRT for Stage I NSCLC.

Local control at 1, 2, 3, and 5 years was respectively 100, 98.2, 98.2, and 77.7% (Figure 2). Three local failures were observed. No statistically significant predictive factor of LC was found (Table 2). PFS at 1, 2, 3, and 5 years was respectively 80, 55.1, 43.7, and 19.7% (Figure 2). Median PFS was 28 months (CI95%: 22–51 months). PFS after lung SBRT was significantly correlated to tumor volume (GTV) in univariate analysis (HR = 1.065, 95%CI = 1.040–1.091, *p* < 0.001) and in multivariate analysis (HR = 1.060, 95%CI = 1.033–1.087, *p* < 0.001), as shown in Table 2. There was a strong trend for significance when we studied mean ipsilateral lung BED_3_ (*p*-value of 0.063; HR = 2.035, 95%CI = 1.000–1.072).

**TABLE 2:** Results of univariate and multivariate analyses for local control, progression-free survival, overall survival and radiation pneumonitis incidence.

	Univariate analysis (LR p BH)				Multivariate analysis (W p)			
	LC	PFS	OS	RP	LC	PFS	OS	RP
Histological type	0.92	0.21	0.18	–	–	–	0.38	–
Pre-treatment SUVmax	0.92	0.96	0.79	–	–	–	–	–
Conformality index	0.92	0.21	0.32	–	–	–	–	–
R50	0.92	0.95	0.85	–	–	–	–	–
GTV	0.92	**<0.001**	**<0.001**	0.91	–	–	–	–
PTV	0.92	**0.003**	**<0.001**	0.89	–	**<0.001**	**0.004**	–
80% prescription isodose volume	0.92	0.21	0.14	–	–	–	–	–
PTV BED_10_								
D_max_	0.92	0.51	0.66	–	–	–	–	–
D_2%_	0.92	0.56	0.66	–	–	–	–	–
D_98%_	0.92	0.80	0.79	–	–	–	–	–
D_min_	0.92	0.80	0.79	–	–	–	–	–
Gender	–	0.86	0.84	–	–	–	–	–
Age	–	0.96	0.46	**0.10**	–	–	–	0.45
Performans status	–	0.56	0.38	–	–	–	–	–
Past medical history of								
Lung surgery	–	0.95	0.79	–	–	–	–	–
Lung radiotherapy	–	0.80	0.66	–	–	–	–	–
Pre-treatment								
FEV1	–	0.80	0.58	**<0.001**	–	–	–	**0.003**
DLCO	–	0.49	0.18	**<0.001**	–	–	0.07	**0.015**
Level of dyspnea (NYHA)	–	0.80	0.65	–	–	–	–	–
Mean lung BED_3_								
Whole lungs	–	0.32	**0.04**	0.09	–	–	**0.01**	–
Ipsilateral lung	–	0.23	**0.04**	**0.04**	–	–	–	–
Whole lungs minus PTV	–	0.32	0.14	**0.02**	–	–	–	0.29
Ipsilateral lung minus PTV	–	0.31	**0.04**	**0.03**	–	–	–	–

*LR p BH: *p*-value with Likelihood ratio adjusted by Benjamini–Hochberg method, W p: *p*-value with Wald test, SUVmax: maximum standardized uptake value, R50: ratio of 50% prescription isodose volume to the PTV, BEDx = biologically effective dose with an α/β ratio of × Gy, FEV1: forced expiratory volume in 1 second, DLCO: diffusing lung capacity for carbon monoxide, NHYA: New York Heart Association. Bold values are those that are statistically significant.*

### Overall Survival

Overall survival at 1, 2, 3, and 5 years was respectively 83, 71.2, 58.1, and 33.2% (Figure 2). Median OS was 49 months. In univariate analysis, significant prognostic factors of OS were GTV (HR = 1.074, 95%CI = 1.047–1.102, *p* < 0.001), PTV (HR = 1.016, 95%CI = 1.010–1.022,*p* < 0.001), mean lung BED_3_ (HR = 1.125, 95%CI = 1.033–1.225, *p* = 0.04), mean ipsilateral lung BED_3_ (HR = 1.060, 95%CI = 1.020–1.103, *p* = 0.04) and mean ipsilateral lung minus PTV BED_3_ (HR = 1.087, 95%CI = 1.023–1.156, *p* = 0.04). Prognostic factors of OS that remained significant in multivariate analysis were mean lung BED_3_ (HR = 1.14, CI95% = 1.03–1.25, *p* = 0.01) and PTV (HR = 1.01, CI95% = 1.0–1.02, *p* = 0.004), as shown in Table 2. Subgroup analysis has been done to study the prognostic role of mean lung BED_3_ in OS. Concerning subgroup analysis of FEV_1_, mean lung BED_3_ remained significantly correlated to OS in multivariate analysis whether for patients with FEV_1_ ≤ 40% (HR = 1.55, 95%CI = 1.12–2.14, *p* = 0.008) or with FEV_1_ > 40% (HR = 1.14, 95%CI = 1.02–1.28, *p* = 0.021). Concerning subgroup analysis of GTV, mean lung BED_3_ remained significantly correlated to OS in multivariate analysis for patients with GTV < mean GTV, i.e., 8.6 cc (HR = 1.34, 95%CI = 1.06–1.69, *p* = 0.015) but not for patients with GTV ≥ mean GTV (HR = 1.02, 95%CI = 0.88–1.18, *p* = 0.78).

**FIGURE 3:**
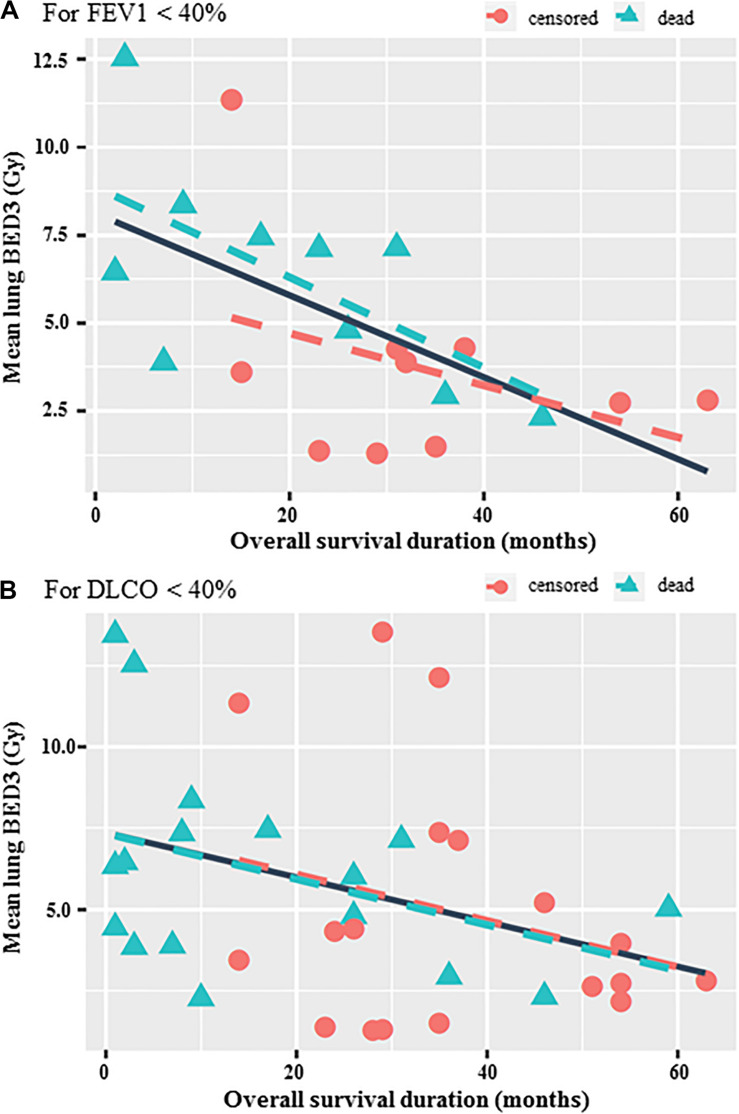
Correlation between OS and mean lung BED3 for patients with FEV1 < 40% (*r* = –0.6, *p* = 0.005) **(A)** and with DLCO < 40% (*r* = –0.36, *p* = 0.033) **(B)**. The solid line shows correlation independently of the censored or dead patients.

**FIGURE 4:**
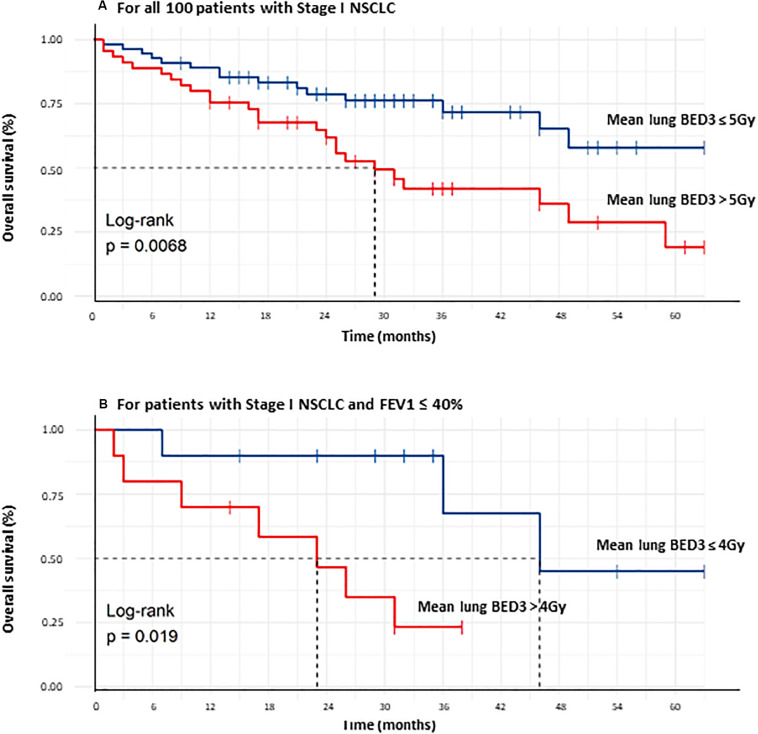
Comparison of overall survival curves of all 100 Stage I NSCLC treated patients between those receiving a mean lung BED_3_ ≤ 5 Gy and those > 5 Gy **(A)**, comparison of overall survival curves of all Stage I NSCLC treated patients with FEV1 ≤ 40% between those receiving a mean lung BED_3_ ≤ 4 Gy and those > 4 Gy **(B)**.

Concerning subgroup analysis for frailty patients with poor baseline pulmonary function, significant negative correlations were observed between OS and mean lung BED_3_ in cases of FEV1 ≤ 40% (*r* = −0.6, *p* = 0.005) and DLCO ≤ 40% (*r* = −0.36, *p* = 0.033) (Figure 3). The best prognostic mean lung BED_3_ threshold identified on the ROC curve in terms of sensitivity and specificity for OS was 5 Gy for the entire population (i.e., 3.6 Gy in 3 fractions or 4.3 Gy in 5 fractions) and reduced to 4 Gy for patients with FEV1 ≤ 40% (i.e., 3 Gy in 3 fractions or 3.5 Gy in 5 fractions). Concerning all the 100 treated patients, a mean lung BED_3_ ≤ 5 Gy was significantly associated with a higher OS (*p* = 0.0068) with a doubling of median OS from 29 months to more than 60 months (not achieved). OS at 1, 2, 3, 4, and 5 years was respectively 89.1, 78.8, 71.8, 65.2, and 58% for a mean lung BED_3_ ≤ 5 Gy rather than 75.6, 61.8, 42, 36, and 19.2% for a mean lung BED_3_ > 5 Gy (Figure 4). Similarly, in patients with poor baseline pulmonary function (i.e., FEV1 ≤ 40%), a mean lung BED_3_ ≤ 4 Gy was significantly associated with a higher OS (*p* = 0.019) with a doubling of median OS from 23 months to 46 months. OS at 1, 2 and 3 years was respectively 90, 90, and 67.5% for a mean lung BED_3_ ≤ 4 Gy rather than 70, 46.7, and 23.3% for a mean lung BED_3_ > 4 Gy (Figure 3).

### Clinical Follow-Up and Toxicities

At the end of follow up, lung SBRT led to 31% Grade ≤ 2 clinically symptomatic radiation pneumonitis (RP), 14% G1 chest wall pain, 7% transient G ≤ 2 radiation dermatitis (4 G1 and 3 G2), 4% rib fractures after a mean period of 28 months (range: 12–45), and 14% G1 chest wall pain. At least 22 patients (22%) died from severe pulmonary insufficiency. Significant predictive factors of clinically symptomatic G ≥ 1 RP were in univariate analysis FEV1 (OR = 0.630, 95%CI = 0.550–0.780, *p* < 0.001), DLCO (OR = 0.750, 95%CI = 0.660–0.859, *p* < 0.001) and mean ipsilateral lung BED_3_ (OR = 0.956, 95%CI = 0.923–0.999, *p* = 0.043). There was a trend towards mean lung BED_3_ (OR = 0.986, 95%CI = 0.998–0.967, *p* = 0.086). In multivariate analysis, FEV1 (OR = 0.929, 95%CI = 0.878–0.971, *p* = 0.003) and DLCO (OR = 0.939, 95%CI = 0.886–0.984, *p* = 0.015) remained significant (Table 2). No significant predictive factor of radiation dermatitis, rib fractures or chest wall pain was found. No other toxicity was observed.

## Discussion

To date, the present study is the first to demonstrate a significant correlation between OS and MLD after Stage I NSCLC SBRT. Moreover, this finding leads us to propose reducing published lung constraints with thresholds of 5 and 4 Gy in mean lung BED_3_ respectively for patients with FEV1 > 40% and FEV1 ≤ 40%.

Our lung SBRT characteristics are in agreement with international guidelines about Stage I NSCLC, especially with Timmerman et al. in the analysis of RTOG 0236 (25). Marginal prescribed dose to the edge of PTV was 54 Gy in three fractions if it met dose constraints. Concerning target volume delineation, we no longer create a CTV since it is not recommended (31). When we started to treat patients in 2012, guidelines were less clear and CTV creation was debated (26). In our study, LC and OS were respectively over 95% and about 60% at the three-year point, which is consistent with previously published studies (1–3, 25). No significant predictive factor of LC was found, probably because only three local failures were observed and adequate BED_10_ was prescribed to the tumor.

Likewise, this study found toxicity in the same proportions as previous reports: 31% clinically symptomatic RP [about 10–20% of patients in literature (range: 0–49% among published studies)] with most patients having asymptomatic Grade 1 pneumonitis (2, 15–17), 14% G1 chest wall pain [10–40% in literature (32)], 7% transient G ≤ 2 radiation dermatitis [12–38% in literature (33)], and 4% rib fractures [0–23% in literature (34)] without other toxicity. No significant predictive factor of chest wall pain, radiation dermatitis or rib fractures was found, probably due to meeting dose constraints (35). Significant predictive factors of RP reported in our study are in agreement with published studies concerning MLD, which is frequently mentioned (15, 17, 19–21). In contrast, baseline pulmonary function (FEV1 and DLCO) was strongly and significantly correlated to RP in univariate and multivariate analyses (18). Thus, the results reported in the present study are demonstrated to be reproducible and may be applied to other studies. As compared to normo-fractionated radiotherapy, MLD corresponds to the most used parameter in predictive risk models pulmonary toxicity due to its simplicity and effectiveness (36, 37). Dose constraint for MLD is often ≤ 15–20, i.e., mean lung BED_3_ ≤ 16–21 Gy (38, 39). Estimated risk of symptomatic RP is 5, 10, 20, 30, and 40% for thresholds of 7, 13, 20, 24, and 27 Gy in MLD, i.e., thresholds of 7, 13, 21, 27, and 30 Gy in mean lung BED_3_ (39).

Radiation pneumonitis is a known important dose-limiting factor in lung cancer radiation therapy. RP is categorized into two interdependent stages: acute RP and late RP (a chronic injury stage known as pulmonary fibrosis) which can theoretically be fatal, especially if pulmonary function is already impaired. The summarized sequence of classic RP is as follows: cellular injury leads to cytokine release, cytokine recruitment of the inflammatory infiltrate causes acute pneumonitis, and the body’s attempt to repair the injury results in pulmonary fibrosis (13). It is accepted that recognition of sporadic RP can be particularly difficult for clinicians because it is rare (≈ 10%) and patients often present with severe dyspnea and/or “out-of-field” radiographic findings that may raise the possibility of other disease process (13). So we can think that RP are probably underestimated in published studies, and especially their exact relationship with the death of patients. This might be one of the explanations of why MLD was a significant prognostic factor of OS in our study.

Limitations of the study were in link with the difficulty to recognize RP particularly in patients with a poor baseline pulmonary function and frequent flares of acute pulmonary insufficiency. It was very difficult to know from what patients were dying and the cause of their pulmonary insufficiency: natural and classic outcome of their comorbidities or especially related to RP? Other limitations were that study was mono institutional and not multicentric, only 100 patients were included which could have led to a lack of power for statistical analyses and data were retrospectively analyzed even if they were prospectively updated. Statistical analysis was robust and there was only one patient lost to follow-up after a period of 10 months after SBRT. Pulmonary heterogeneity, which may be a reason for false interpretation, has been taken into account by using the Monte Carlo algorithm for DCA plans and the AAA algorithm for VMAT plans. Various dose prescriptions and fractionations have been studied with the linear-quadratic model with an α/β ratio of 3 Gy which was the best method for converting the physical lung dose to predict RP (24).

Concerning PFS, PFS after lung SBRT was significantly correlated to tumor volume (GTV) in univariate and in multivariate analysis. We did not find mean lung BED_3_ as a significant prognostic factor of PFS in univariate analysis. One of the explanations might be a lack of power of our study because the lower threshold value of the 95%CI was very close to 1 (0.988). Interestingly, there was therefore a strong trend for significance when we studied mean ipsilateral lung BED_3_ (*p*-value of 0.063; HR = 2.035, 95%CI = 1.000–1.072).

Concerning OS, it is widely agreed that more patients die of comorbidities than of tumor progression since LC is excellent, over 95% at three years. Therefore, significant published prognostic factors to date mostly relate to comorbidities [age (4), gender (5), performance status (4, 6), platelet-to-lymphocyte ratio (8, 10), and pretreatment immune parameters (10)] if the prescribed dose is sufficiently high (11). However, no relationship between toxicity and OS is demonstrated to date. Many patients treated with lung SBRT die from severe pulmonary insufficiency, but it is not easy to distinguish whether the main cause is the natural course of pulmonary or cardiovascular disease or if SBRT lung toxicity might have worsened the situation. Severe RP is probably under-evaluated in literature. It is the main toxicity factor after lung SBRT; furthermore, MLD seems to be the strongest and most reproducible dosimetric parameter of RP (15, 17, 19–21). In addition, tumor volume is a reproducible and frequently reported significant prognostic factor of OS (5–7). We can also surmise that tumor volume is linked to MLD and that patients could die from a higher received MLD than GTV because LC is high. For this reason, we have made the choice to study the correlation between OS and MLD, which we have demonstrated to be significant in multivariate analysis for all patients (HR = 1.14, CI95% = 1.03–1.25, *p* = 0.01) and in subgroup multivariate analysis, whether for patients with FEV_1_ ≤ 40% (HR = 1.55, 95%CI = 1.12–2.14, *p* = 0.008) or with FEV_1_ > 40% (HR = 1.14, 95%CI = 1.02–1.28, *p* = 0.021) or with GTV < mean GTV, i.e., 8.6 cc (HR = 1.34, 95%CI = 1.06–1.69, *p* = 0.015). GTV and FEV_1_ cannot be modified while the prescribed dose can be adjusted.

Moreover, we showed in our study a significant correlation between OS and MLD with a significant threshold value of 5 Gy for BED_3_, i.e., 3.6 Gy in three fractions. A significant cut-off at 4–4.7 Gy in three fractions is reported in literature concerning the probability of RP after lung SBRT (17, 19). So our threshold value of 3.6 Gy in three fractions is not very different from that published for symptomatic RP, which enables us to assume that the threshold value of 5 Gy for OS may be related to RP. Thresholds values may have to be reduced to 5 Gy (i.e., 3.6 Gy in 3 fractions or 4.3 Gy in 5 fractions) and 4 Gy (i.e., 3 Gy in 3 fractions or 3.5 Gy in 5 fractions) in mean lung BED_3_ respectively for patients with FEV1 > 40% and FEV1 ≤ 40%, as our study suggests, to have an impact on OS. These two thresholds values are totally in agreement because we may have to be more careful to treat frailty patients and probably we may have to reduce prescribed dose for these patients. We therefore recommend using effective algorithms that take into account pulmonary heterogeneity and limiting PTV irradiated volume to a minimum by reducing margins (not creating a CTV or using gating or tracking techniques). This is particularly crucial for frailty patients with poor baseline pulmonary function (FEV1 ≤ 40%).

## Conclusion

In summary, our study demonstrates a significant and strong correlation between OS and mean lung BED_3_, confirmed in univariate and multivariate analysis in all patients, in subgroup analysis and in survival curves analysis. Higher mean lung BED_3_ is always strongly and significantly associated with a poorer OS. Moreover, significant mean lung BED_3_ threshold values have here been shown to correlate with OS: 5 Gy for the entire population (i.e., 3.6 Gy in 3 fractions or 4.3 Gy in 5 fractions) and 4 Gy for patients with FEV1 ≤ 40% (i.e., 3 Gy in 3 fractions or 3.5. Gy in 5 fractions). Stay below these threshold values significantly enabled a doubling of median OS.

## Data Availability Statement

The datasets generated for this study are available on request to the corresponding author.

## Ethics Statement

Study ethics approval was obtained on 06 December 2019 (CECIC Rhône-Alpes-Auvergne, Grenoble, IRB 5921).

## Author Contributions

GD and IM analyzed the data. GD wrote and revised the manuscript. JB, VC, VD, ML, and AB-C also revised the manuscript. All authors have actively participated in the data acquisition, commented, and approved the final version of the manuscript.

## Conflict of Interest

The authors declare that the research was conducted in the absence of any commercial or financial relationships that could be construed as a potential conflict of interest.

## Abbreviations

18F-FDG PET: 18-F FluoroDeoxyGlucose Positron Emission Tomography
BED: biologically effective dose
CBCT: cone beam CT
CI: conformality index ratio of 80% prescription isodose volume to the PTV
CT: computed tomography
CTV: clinical target volume
D2cm: maximum dose 2 cm from the PTV in any direction
DCA: dynamic conformal arcs
DLCO: diffusing lung capacity for carbon monoxide
FEV1: forced expiratory volume in 1 second
GTV: gross tumor volume
Gy: Gray
ITV: internal target volume
LC: local control
LINAC: LINear ACcelator
MLD: mean lung dose
MRI: magnetic resonance imaging
NCI-CTCAE: National Cancer Institute’s Common Toxicity Criteria for Adverse Events
NSCLC: non-small-cell lung cancer
NYHA: New York Heart Association
OS: overall survival
PFS: progression-free survival
PS: performance status
PTV: planning target volume
R50: ratio of 50% prescription isodose volume to the PTV
RECIST: Response Evaluation Criteria in Solid Tumors
ROC: receiver operating characteristics
RP: radiation-induced pneumonitis
RTOG: Radiation Therapy Oncology Group
SBRT: stereotactic body radiation therapy
SUVmax: maximum standardized uptake value
TPS: treatment planning systems
VMAT: volumetric modulated arc therapy.
